# Comparative study of three treatment approaches on overall survival and treatment response in nasopharyngeal carcinoma patients: network meta-analysis of RCTs (4221 patients)

**DOI:** 10.3389/fonc.2026.1748308

**Published:** 2026-07-02

**Authors:** Jun Hu, Li Haojie

**Affiliations:** 1 Department of Otolaryngology, Taizhou Hospital of Zhejiang Province affiliated to Wenzhou Medical University, Taizhou, China; 2 School of Exercise and Health, Shanghai University of Sport, Shanghai, China

**Keywords:** Induction chemotherapy, nasopharyngeal carcinoma patients, network meta-analysis, overall survival, targeted therapy

## Abstract

**Objective:**

Nasopharyngeal carcinoma (NPC) is a malignant tumor with significant disease burden. Currently, radiotherapy-based multimodal therapy remains the primary treatment strategy for NPC, yet consensus on the relative efficacy of induction chemotherapy, targeted therapy, and radiotherapy remains elusive. This study aims to systematically compare the relative efficacy of induction chemotherapy, targeted therapy, and radiotherapy on overall survival and treatment response using network meta-analysis, thereby providing evidence-based guidance for clinical decision-making.

**Methods:**

Following the PRISMA-NMA and Cochrane Manual guidelines, we systematically searched six databases (PubMed, Embase, Web of Science, Cochrane Library, EBSCO, and CNKI) for relevant randomized controlled trials (RCTs) published between January 1998 and June 2025. Two researchers independently conducted literature screening, data extraction, and risk of bias assessment. A total of 12 randomized controlled trials were ultimately included. Traditional meta-analysis and heterogeneity assessment were performed using RevMan 5.3 software. A network meta-analysis was conducted using STATA 17.0 software (Stata Corp LLC, College Station, TX, USA) based on a frequency framework, with interventions ranked by cumulative ranked probability area under the curve (SUCRA). Publication bias was evaluated using a corrected funnel plot.

**Results:**

Network meta-analysis showed that while TD ranked first in the probability of being the best treatment for OS (SUCRA = 98.1%), only IC demonstrated a statistically significant survival benefit compared to the control group (HR = 0.37, 95% CI: 0.02, 0.71). Regarding PFS, TD ranked first (SUCRA = 82.2%, HR = -0.68, 95% CI: -2.59, 1.23), although no intervention demonstrated a statistically significant benefit compared to the control group. In terms of ORR, IC had the highest probability (SUCRA = 71.6%, OR = 0.24, 95% CI: -2.18, 2.66), with no statistically significant differences among the interventions. Regarding CRR, TD showed the greatest advantage (SUCRA = 98.0%, OR = 0.26, 95% CI: 0.12, 0.58).

**Conclusion:**

The network meta-analysis results indicate that induction chemotherapy offers the greatest advantage in improving overall survival for nasopharyngeal carcinoma patients, while targeted drugs perform best in reducing cumulative recurrence risk. No statistically significant differences were observed among the three interventions for progression-free survival or objective response rate. Radiotherapy alone did not demonstrate significant benefit across any outcome measures. This study provides important evidence-based support for individualized treatment decisions in nasopharyngeal carcinoma.

**Systematic Review Registration:**

https://www.crd.york.ac.uk/prospero/, identifier CRD420251178558.

## 1 Introduction

Nasopharyngeal carcinoma (NPC) is a malignant tumor originating from the epithelium of the nasopharynx, characterized by distinct geographical and biological features (1). Unlike many other head and neck cancers, nasopharyngeal carcinoma exhibits a highly concentrated regional distribution, with the highest incidence rates exceeding 20–30 cases per 100,000 person-years in populations of southern China, Southeast Asia, North Africa, and certain Arctic regions (2). This significant geographic clustering is closely associated with specific etiological factors, including Epstein-Barr virus infection, genetic susceptibility, and environmental exposures (3). Most patients are diagnosed at a locally advanced stage, and the WHO histological subtypes II and III, which predominate in endemic regions, are associated with varying treatment responses and prognoses (4). Patients commonly present with symptoms such as nasal obstruction, epistaxis, hearing loss, and headaches, which stem from both the primary tumor and treatment-related toxicities, thereby severely impairing quality of life and social functioning (5). The high incidence in endemic regions, late presentation, and prolonged multimodal treatment regimens impose a significant socioeconomic burden on affected populations and healthcare systems. Therefore, optimizing treatment strategies to improve survival outcomes and minimize long-term morbidity remains a key priority in clinical practice and research.

Within the efficacy evaluation framework for NPC, overall survival (OS) is regarded as the gold standard for assessing the long-term value of treatment regimens, directly reflecting the ability of therapeutic approaches to prolong patient life (6). Concurrently, treatment response, encompassing objective response rate (ORR) and progression-free survival (PFS), serves as a crucial indicator for measuring the immediate efficacy of treatments and tumor sensitivity (7). Evidence suggests that aggressive and effective treatment significantly improves patient survival outcomes. However, the pathophysiological mechanisms of NPC are complex, involving factors such as Epstein-Barr virus infection, genetic susceptibility, and environmental influences, leading to substantial individual variation in responses to identical treatment regimens (8, 9). Numerous studies have investigated prognostic factors associated with survival and treatment response, evaluating the efficacy of various therapeutic approaches. These studies consistently affirm that the choice of treatment strategy is a decisive factor influencing patients’ long-term survival and quality of life. Nevertheless, ongoing debate persists regarding which combination or sequential treatment regimen yields the greatest survival benefit and optimal tumor response (10).

Currently, radiotherapy-based comprehensive treatment remains the standard approach for locally advanced NPC. With advancements in medical technology, treatment modalities have evolved from radiotherapy alone to a multimodal system integrating chemotherapy, targeted therapy, and other modalities centered around radiotherapy (11). Among these, concurrent chemoradiotherapy (CCRT) is the established standard of care for locoregionally advanced NPC, and adjuvant chemotherapy has also been investigated as an additional strategy to reduce distant failure. To further enhance efficacy, the application of induction chemotherapy (IC) and targeted therapy (TD) has become increasingly widespread. Induction chemotherapy, administered prior to definitive radiotherapy, aims to eliminate micro metastases, reduce primary tumor volume, and create favorable conditions for subsequent radiotherapy (12). Landmark phase III trials have established gemcitabine plus cisplatin (GP) as a cornerstone induction regimen (13), demonstrating significant improvements in tumor control and overall survival. Targeted therapies studied in NPC include anti-PD-1 immune checkpoint inhibitors, such as toripalimab, camrelizumab, and pembrolizumab, as well as bevacizumab; these agents enhance anti-tumor immunity or inhibit angiogenesis when combined with chemoradiotherapy (14). The widespread adoption of intensity-modulated radiation therapy has further improved the precision of radiotherapy, enhanced local tumor control, reduced radiation-induced damage to surrounding organs, and has been associated with improved overall survival in nasopharyngeal carcinoma patients (15, 16) Previous studies have provided evidence supporting the efficacy of each approach. For instance, some research confirms the benefit of IC in improving progression-free survival, while other studies indicate that adding targeted agents to CCRT further enhances survival in specific patient populations (17).

Despite the theoretical advantages and clinical evidence supporting IC, TD, and radiotherapy (RT), no clear consensus exists regarding which strategy delivers optimal overall survival and the highest treatment response rate for NPC patients. Existing randomized controlled trials (RCTs) directly comparing these regimens are limited in number and often restricted to head-to-head comparisons between two strategies, lacking a comprehensive evaluation that integrates all three mainstream approaches within a unified analytical framework. Furthermore, discrepancies in patient populations, study designs, and statistical power among these studies often yield conflicting conclusions, resulting in a fragmented evidence base that complicates clinical decision-making. Notably, concurrent chemoradiotherapy is established as the standard control in many RCTs due to its foundational role in current treatment guidelines. Therefore, there is an urgent need for an advanced statistical methodology capable of integrating both direct and indirect evidence to systematically evaluate and rank the relative efficacy of these three key treatment strategies against this common benchmark.

To address this gap, this study employed a network meta-analysis (NMA) to systematically integrate and quantitatively analyze relevant randomized controlled trials (RCTs). By conducting indirect comparisons, the relative efficacy of IC, TD, and RT was evaluated simultaneously within a single model, and probability rankings were provided. By comprehensively examining long-term survival (OS) and short-term treatment responses (PFS, ORR, CRR), this study aims to establish a multidimensional efficacy evaluation system. The findings are expected to provide evidence for clinical decision-making and guideline development, fill gaps in existing research, guide future research directions, and ultimately contribute to improving survival outcomes and quality of life for nasopharyngeal carcinoma patients.

## 2 Methods

This study was guided by the Preferred Reporting Items for Systematic Reviews and Meta-Analysis (PRISMA) checklist for NMAs and the Cochrane Handbook for the Systematic Review of Interventions (18). Registration number: CRD420251178558.

### 2.1 Data sources

We conducted systematic searches in PubMed, Embase, Web of Science, Cochrane, EBSCO, and China National Knowledge Infrastructure (CNKI). Two researchers (JH, HL) independently performed the selection of included studies. Searches in PubMed and Cochrane utilized terms from the Medical Subject Headings (MeSH) database. Embase searches employed terms from Emtree, while CNKI searches combined subject headings with free-text keywords. Additionally, reference lists of relevant articles were manually screened to identify other potentially eligible studies. The search period spanned from January 1998 to June 2025, restricting studies to human research published in Chinese or English, with Chinese studies limited to core journals.

The search strategy adhered to the evidence-based medicine PICOS framework: (P) Population: Patients with stage II–IVB nasopharyngeal carcinoma; (I) Intervention: Targeted drugs, induction chemotherapy, radiotherapy; (C) Control group: Concurrent chemoradiation therapy; (O) Outcomes: OS (Overall survival), PFS (Progression-free survival), ORR (Objective response rate), CRR (Cumulative recurrence rate); (S) Study type: RCTs. The full PubMed search strategy, including the number of hits and search date, is provided in **Supplementary 1**.

### 2.2 Study selection

Medical search terms were used to conduct searches in PubMed and Cochrane. To ensure comprehensiveness and accuracy, reference lists of relevant articles were manually screened to identify additional studies potentially meeting the criteria. Literature related to induction chemotherapy, targeted drugs, and nasopharyngeal carcinoma patients was systematically retrieved.

Following the initial literature acquisition, a rigorous screening process was implemented. First, EndNote software was used to automatically detect duplicate records, eliminating potential duplicates arising from differences in search strategies or data sources across databases. Subsequently, manual review of titles and abstracts further removed duplicates not automatically identified during screening, ensuring the selected literature was unique and representative. The remaining articles underwent more rigorous examination. The following types of studies were primarily excluded: research involving non-nasopharyngeal carcinoma patient populations, studies that did not evaluate relevant indicators, and studies that did not employ targeted drugs, induction chemotherapy, or radiotherapy alone as interventions. Additionally, review articles, conference abstracts, animal studies, research protocols, case reports, retrospective studies, and book chapters were excluded. These often lack sufficient original data or scientific rigor, making it difficult to provide concrete analyses of intervention effects and conclusions. These stringent literature screening criteria ensured that the ultimately included studies provided high-quality evidence supporting this research, further enhancing its scientific validity and credibility.

### 2.3 Eligibility criteria

We included randomized clinical trials in people with confirmed nasopharyngeal carcinoma comparing the effects of different treatments.

Studies were eligible for inclusion if they met the following criteria: (1) were randomized controlled trials (RCTs); (2) involved adult nasopharyngeal carcinoma patients with stage II to IVB disease; (3) had complete outcome data for at least one of the following outcomes: OS, PFS, ORR or CRR; (4) The experimental group received one of the following interventions: targeted therapy using drugs such as toripalimab or bevacizumab; induction chemotherapy with drugs like gemcitabine and cisplatin administered before concurrent chemoradiotherapy; or radiotherapy alone. The control group received radiotherapy or concurrent chemoradiotherapy. see **Table 1**. (5) Measured at least one of the following indicators: OS (Overall Survival); PFS (Progression-Free Survival); ORR (Objective Response Rate); CRR (Cumulative Recurrence Rate).

**Table 1:** Classification criteria and definitions for treatment regimens.

Intervention methods	Abbreviation	Definition
Induction Chemotherapy	IC	Trials evaluating the addition of induction chemotherapy to definitive radiotherapy or concurrent chemoradiotherapy as the primary incremental intervention
Targeted Drugs	TD	Trials evaluating the addition of targeted agents (e.g., anti-PD-1 inhibitors, bevacizumab) to chemotherapy, radiotherapy, or concurrent chemoradiotherapy as the primary incremental intervention
Radiotherapy Alone	RT	Trials evaluating radiotherapy without concurrent chemotherapy, compared against radiotherapy-based multimodal regimens

Studies were excluded if they: (1) were non-RCTs; (2) were animal studies, review articles, conference reports, case reports, letters, or duplicated publications; (3) lacked full-text availability; (4) had incomplete experimental results or unobtainable data metrics; (5) failed to report relevant indicators of interest to this study; (6) included patients with other oropharyngeal diseases besides nasopharyngeal carcinoma.

### 2.4 Data collection

Two researchers (JH, HL) imported the collected literature into EndNote 20 software according to the search strategy and screened the retrieved documents. First, duplicate records were excluded. Then, titles and abstracts were reviewed for preliminary screening. The remaining documents underwent further screening through detailed full-text reading based on inclusion and exclusion criteria. Subsequently, two researchers (JH, LH) cross-checked each other’s screening results. If agreement was reached, the study was included; if disagreement arose, a third researcher (XY) mediated the discussion until consensus was achieved before final inclusion.

For eligible studies, two trained researchers (JH, HL) independently extracted data from the included publications using standardized data extraction forms and assessed risk of bias. Extracted data primarily included: (1) basic study information (first author, publication year, country, etc.); (2) participant demographics (number, age, and gender in intervention and control groups); (3) intervention details (type, duration, frequency); (4) Outcome measures (number of events and total participants; selected primary outcomes included measures assessing overall survival, progression-free survival, objective response rate, and cumulative recurrence rate). For studies presenting results in graphical formats without numerical summaries, validated graph digitizing tools (GetData 2.22) were used to extract numerical data for analysis. Where necessary, we contacted article authors to obtain information.

### 2.5 Risk of bias assessment

Using the Cochrane Risk of Bias Assessment Tool version 5.1 (which includes seven domains: random sequence generation, allocation concealment, blinding of participants and personnel, blinding of outcome assessors, incomplete data on outcomes, selective reporting, and other bias), two researchers (XL, HL) assessed the risk of bias (ROB) for all eligible studies. Risk assessment analysis was performed using Review Manager 5.3 (Cochrane Nordic, Denmark) (19), with each domain rated as unclear, low risk, or high risk. Based on these assessments, we classified the overall risk of bias for each study as follows: (1) Low ROB: No domains rated high risk; may have up to two domains rated unclear; (2) Moderate ROB: At least one domain rated high risk, or no high-risk domains but more than three rated unclear; (3) High ROB: All other scenarios not covered above.

### 2.6 Statistical analysis

Data analysis was performed using RevMan software version 5.3 (Cochrane Collaboration, Oxford, UK) (18). For survival outcomes (including overall survival [OS] and progression-free survival [PFS]), treatment effects were assessed using hazard ratios (HR) and their 95% confidence intervals (CI). The hazard ratio, which compares the instantaneous risk of events (such as death or disease progression) across the entire follow-up period between intervention groups, is the most appropriate and conventional measure of effect for such outcomes in oncology. This is because it simultaneously accounts for both the number of events and the time at which they occur. For dichotomous outcome measures (including objective response rate [ORR] and cumulative recurrence rate [CRR]), the odds ratio (OR) and its 95% confidence interval (CI) were used to assess efficacy. The OR was selected as the primary effect measure due to its superior statistical properties in meta-analysis models, including mathematical symmetry and stability. Cochrane guidelines recommend the OR as the default measure because it demonstrates greater robustness in complex statistical models. Inter-trial heterogeneity was assessed using the Cochrane Q test and I² statistic, which quantifies the proportion of total variation attributable to heterogeneity rather than random factors. Heterogeneity was categorized as low (I² 25%-50%), moderate (I² 50%-75%), or high (I² >75%). The meta-analysis employed a random-effects model. This study conducted a network meta-analysis using STATA 17.0 software (Stata Corp LLC, College Station, Texas, USA). The relationships between different drug treatments were visualized using a network diagram, in which the lines connecting nodes represent direct comparisons between non-invasive methods. Node size and line thickness are proportional to the number of studies included in each comparison. This graphic intuitively illustrates the relative strength of each intervention and its position within the network. Additionally, the constructed network contribution plot further quantifies the contribution of each direct comparison to the overall network, aiding in the analysis of the influence of each intervention within the network. Furthermore, to assess publication bias in the studies, adjusted funnel plot analyses were conducted for the primary outcome measures. Finally, the probability of each intervention being the best option was calculated using the Sum of Cumulative Ranked Area (SUCRA) method. Sensitivity analyses were conducted using the leave-one-out method to assess the influence of individual studies on the pooled effect estimates.

## 3 Result

### 3.1 Study selection

**Figure 1:**
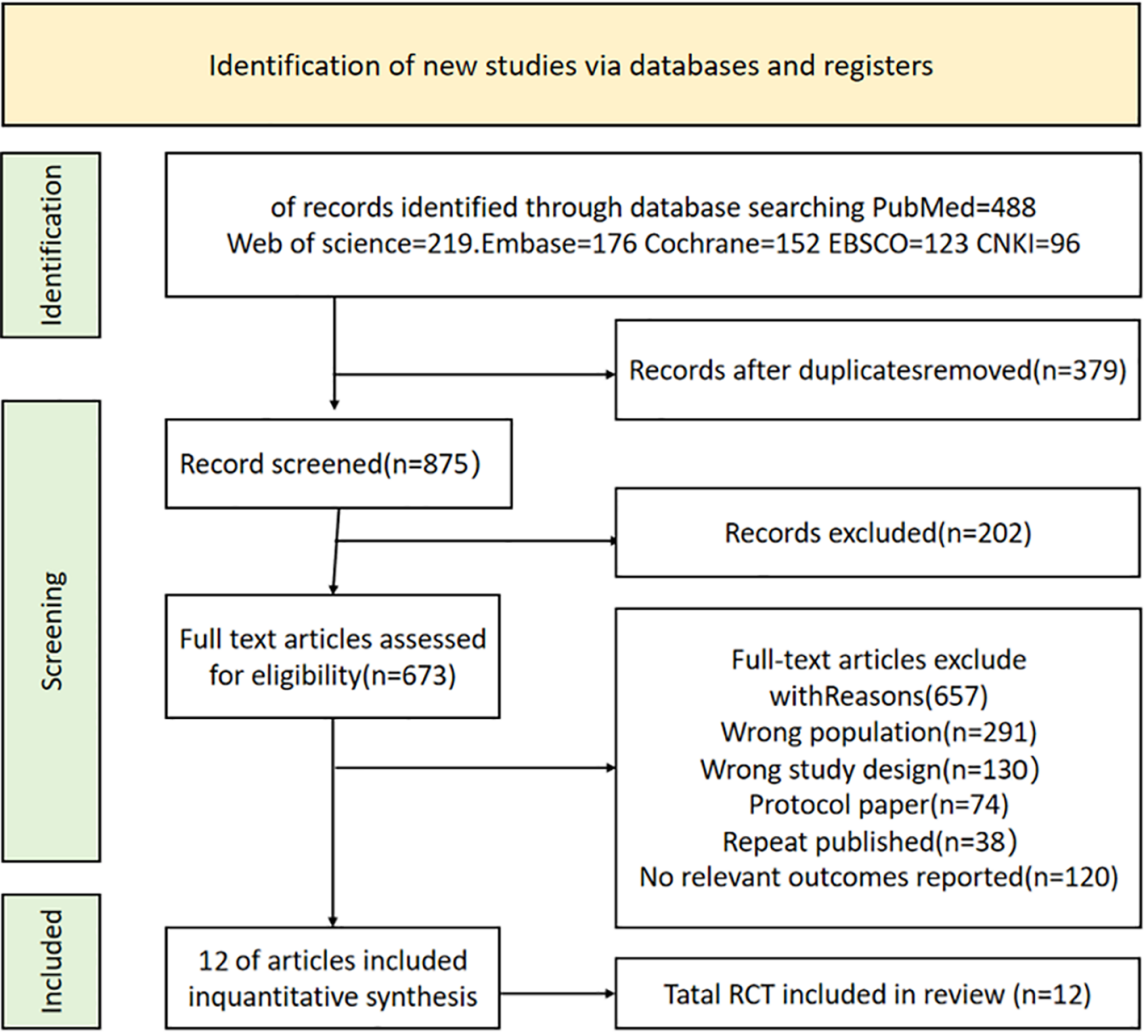
Literature search flowchart.

A total of 1,254 potentially relevant publications were identified through electronic searches. After removing 379 duplicate records, 875 articles remained for screening. Screening of titles and abstracts resulted in the exclusion of 202 publications. Full-text reviews based on inclusion criteria further excluded 653 ineligible publications, ultimately yielding 12 included studies. The literature selection process is illustrated in **Figure 1**.

### 3.2 Literature characterization

A total of 12 studies were ultimately included. The table summarizing the key characteristics of all included studies is presented in **Table 2**. This systematic review and network meta-analysis encompassed studies published from 1998 to 2024, conducted in Singapore, Greece, Romania, the United States, Thailand, Malaysia, Indonesia, and China. The experimental groups comprised 536 nasopharyngeal carcinoma patients receiving targeted drug therapy, 908 undergoing induction chemotherapy, and 696 undergoing radiotherapies. The control group included 2081 participants. Participants ranged in age from 25 to 80 years, totaling 4221 trial participants. Detailed characteristics are presented in **Table 1**. Across 16 randomized controlled trials, three drug therapies (TD, IC, RT) were compared with concurrent chemoradiotherapy interventions. Induction chemotherapy was most frequently used (37.5%), followed by TD and RT (both 31.25%). The median follow-up duration ranged from 15.6 to 82.6 months. Patients with nasopharyngeal carcinoma stages II to IVB were included. In targeted therapy studies, toripalimab, pembrolizumab, camrelizumab, and bevacizumab served as primary interventions. Induction chemotherapy studies predominantly employed platinum-based combination regimens. Radiotherapy studies compared the efficacy of radiotherapy alone versus concurrent chemoradiotherapy. **Table 1** details the intervention measures, drug dosages, radiotherapy modalities, and study designs for each study.

**Table 2:** Basic features of the included.

Author	Group	Year	N	(T/C)	Source	Stage	Median follow up (m)	Targeted drugs	Chemotherapy	Induction chemotherapy	Radiation therapy
Liu (19)	TD	2024	150	100/50	China	AJCC III-IVA	37.8	240 mg toripalimab	Cisplatin 100 mg/m2	None	IMRT:2.1gy/f to 54-70gy
Zhang (13)	IC	2022	480	242/238	China	AJCC/UICC III–IVB	69.8	None	Gemcitabine/Cisplatin	Gemcitabine/Cisplatin	IMRT
Liu (20)	IC	2022	104	52/52	China	AJCC III	33.8	None	capecitabine 1000 mg/m2	cisplatin [60 mg/m2 paclitaxel [150 mg/m2	None
Li (21)	IC	2022	238	52/52	China	AJCC III	33.8	None	Paclitaxel 150 mg/m2 Cisplatin 60 mg/m2 Capecitabine 1000 mg/m2	Cisplatin 100 mg/m2 Fluorouracil 800 mg/m2	IMRT
Qi (22)	IC	2019	476	238/238	China	AJCC III	82.6	None	Cisplatin 80 mg/m²	Cisplatin 80 mg/m² Fluorouracil 800 mg/m²	IMRT/2DRT:2.0–2.33 Gy/f
Lv (23)	IC	2021	502	252/250	China	UICC/AJCC III–IVB	75.3	None	Lobaplatin 30 mg/m² Cisplatin 100 mg/m²	Lobaplatin 30 mg/m Fluorouracil 800 mg/m²	IMRT:2.12–2.33 Gy/f to 54-70
Fountzilas G (24)	IC	2012	141	72/69	Greece	AJCC 2002 IIB-IVB	55	None	Cisplatin 40 mg/m²	Cisplatin 75 mg/m² Epirubicin 75 mg/m² Paclitaxel 175 mg/m²	3D-CRT/2D-RT: 1.8–2.0 Gy/f to 48–78 Gy
Tang (25)	RT	2022	341	172/169	China	AJCC II/T3N0	46	None	Cisplatin 100 mg/m²	None	IMRT:2.0–2.2 Gy/f to 68-70GY
Lee (26)	RT	2010	348	172/176	China	AJCC/UICC III-IVB	70.8	None	Cisplatin 100 mg/m² Fluorouracil 1000 mg/m²	None	CFRT:2.0 Gy/f to 50–68 Gy
Chen (27)	RT	2011	230	116/114	China	AJCC II–III	60	None	Cisplatin 30 mg/m²	None	2D-RT:2 Gy/f to 50–70 GY
M Al-Sarraf (28)	RT	1998	147	69/78	USA	AJCC III–IV	32.4	None	Cisplatin 100 mg/m²	None	IMRT :1.8–2.0 Gy/to 70GY
Chua (29)	RT	1998	334	167/167	China	Ho’s Stage III - IV	30	None	Cisplatin:60 mg/m² Epirubicin:110 mg/m²	Cisplatin:60 mg/m² Epirubicin:110 mg/m²	XRT: 2.1 Gy/f to 54–70 Gy

### 3.3 Risk of bias assessment

**Figure 2:**
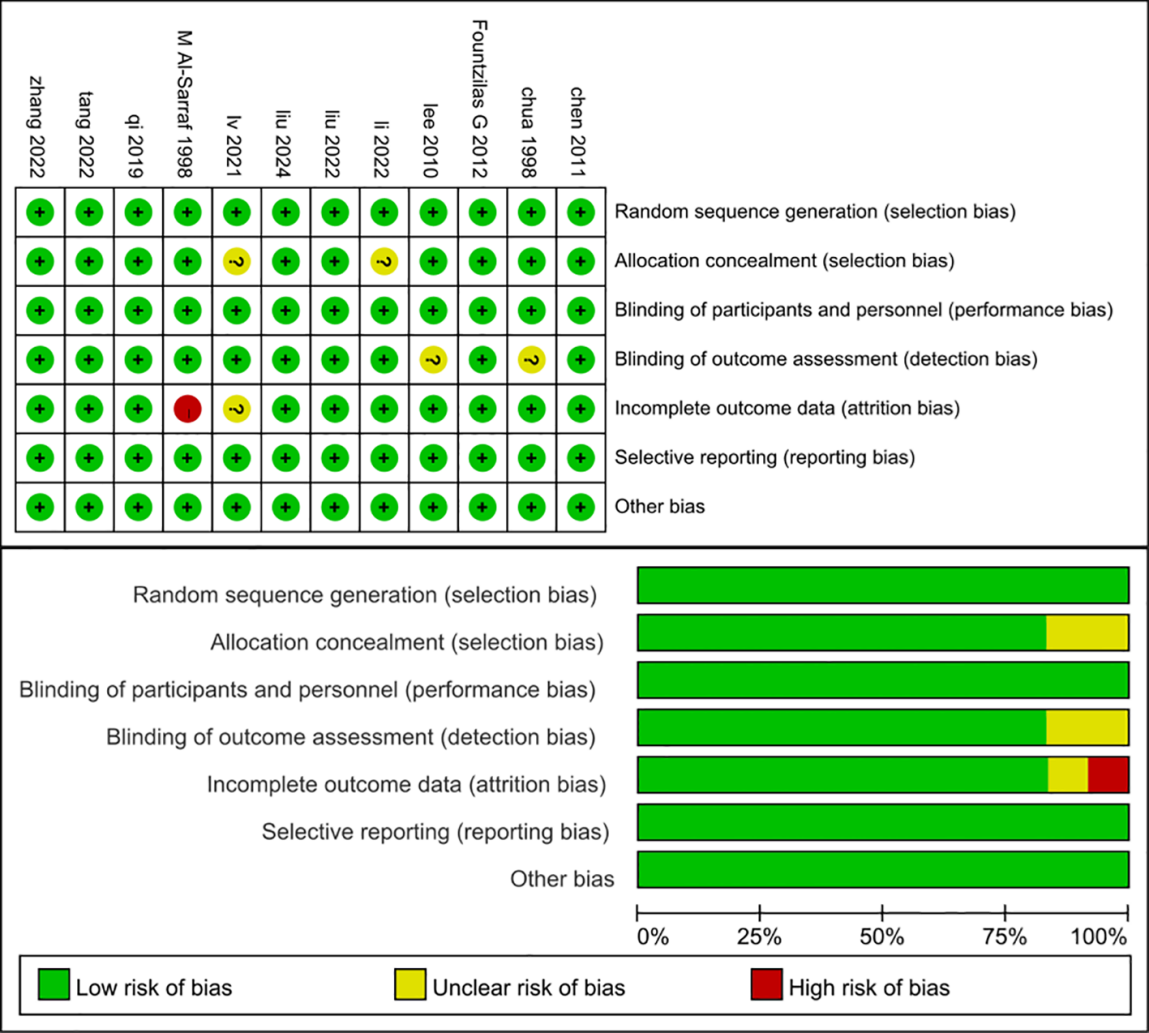
Risk offset map.

**Table 3** and **Figure 2** provide detailed information on the ROB assessment for each study. Among these 12 articles, all 12 mentioned random allocation, with 12 describing the random allocation method and 10 describing allocation concealment; 11 reported blinding of participants and personnel; 10 reported blinding of outcome assessment; 11 studies demonstrated a low risk of incomplete outcome data; 12 studies showed a low risk of selective reporting; and all 12 articles were free of other biases. Overall, the risk of bias across all 12 articles was assessed as low.

**Table 3:** Evaluation results of literature quality risk bias of included studies.

Inclusion of literature	Random sequence generation	Allocation concealment	Blinding of participants and personnel	Blinding of outcome assessment	Incomplete outcome data	Selective reporting	Other bias
Liu (19)	L	L	L	L	L	L	L
Zhang (13)	L	L	L	L	L	L	L
Liu (20)	L	L	L	L	L	L	L
Li (11)	L	u	L	L	L	L	L
Qi (22)	L	L	L	L	L	L	L
Lv (23)	L	u	L	L	u	L	L
Fountzilas G (24)	L	L	L	L	L	L	L
Tang (25)	L	L	L	L	L	L	L
Lee (26)	L	L	L	u	L	L	L
Chen (27)	L	L	L	L	L	L	L
M Al-Sarraf (28)	L	L	L	L	h	L	L
Chua (29)	L	L	L	u	L	L	L

L, low risk of bias; U, unclear risk of bias; H, high risk of bias.

### 3.4 Direct pairwise meta-analyses

**Figure 3:**
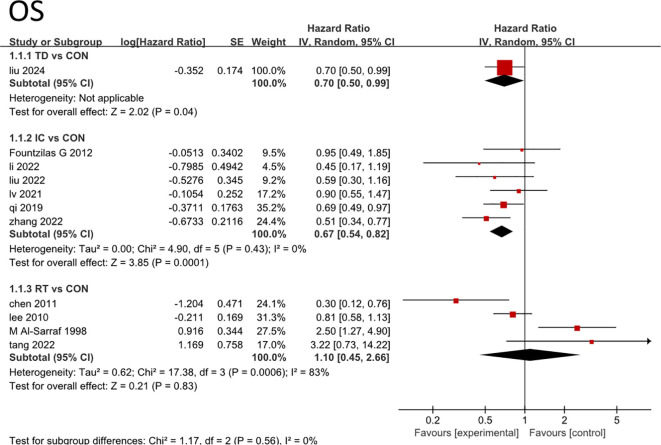
Forest of overall survival.

The meta-analysis of OS included multiple studies and systematically assessed the impact of various interventions on survival outcomes. The results showed that for TD, only one study was included (19), with a pooled HR of 0.70 (95% CI: 0.50–0.99, P = 0.04). Although this result is statistically significant, given that the evidence is derived from a single study, the robustness and generalizability of the findings are limited, and caution is warranted in their interpretation. The IC group included a total of 6 studies, with an HR of 0.67 (95% CI: 0.54–0.82, P = 0.0001), indicating that IC significantly improves overall survival. There was no heterogeneity among the studies in this group (I² = 0%), and the results were robust. The RT group included 4 studies, with an HR of 1.10 (95% CI: 0.45–2.66, P = 0.83), showing no significant survival benefit, and high heterogeneity among the studies (I² = 83%). Subgroup analysis revealed no statistically significant differences among the three subgroups (P = 0.56, I² = 0%), suggesting that the relative efficacy of the interventions did not differ significantly overall, although there were marked differences in the direction and consistency of effects within each group (**Figure 3**).

**Figure 4:**
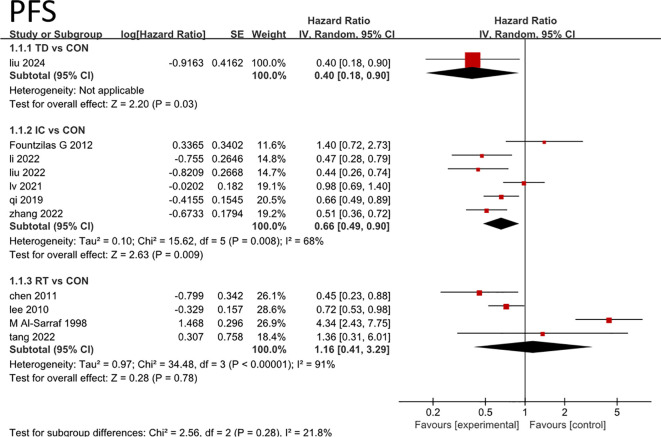
Forest of progression-free survival.

The meta-analysis on PFS included multiple studies and systematically assessed the impact of different interventions on disease progression. The forest plot results showed that for TD, only one study was included, with a pooled HR of 0.40 (95% CI: 0.18–0.90, P = 0.03), suggesting that TD significantly reduces the risk of disease progression compared to the control group. However, as this result is based on a single study, the robustness of the evidence is limited, and caution should be exercised in its interpretation. For IC, a total of 6 studies were included, with a pooled HR of 0.66 (95% CI: 0.49–0.89, P = 0.009), indicating that IC significantly improves progression-free survival. There was moderate heterogeneity among the studies in this group (I² = 68%, P = 0.008), suggesting some variation in study results; interpretation of the findings should be informed by an analysis of the sources of heterogeneity. For RT, four studies were included, yielding a pooled HR of 1.16 (95% CI: 0.41–3.29, P = 0.78). No significant benefit in disease progression was observed, and there was extremely high heterogeneity among the studies (I² = 91%, P < 0.00001), indicating a high degree of inconsistency in the direction and magnitude of the effects of radiotherapy across different studies. Subgroup analysis revealed no statistically significant differences in efficacy among the three subgroups (P = 0.28, I² = 21.8%), suggesting that, in this analysis, there were no significant overall differences in the relative efficacy of the various intervention types, but there were marked differences in the consistency of effects within each group (**Figure 4**).

**Figure 5:**
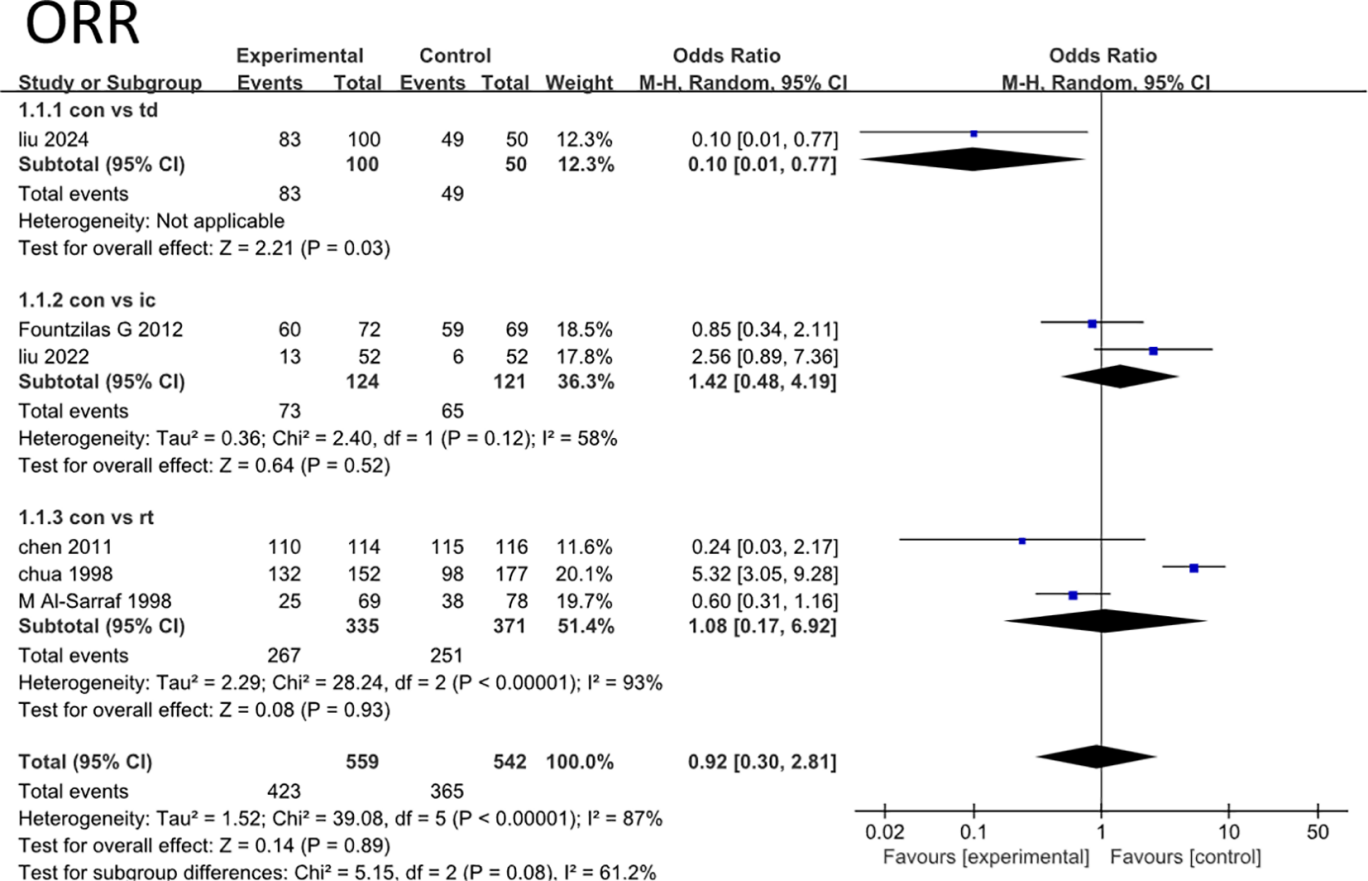
Forest of objective response rate.

In the meta-analysis of ORR, the forest plot results showed that in the TD group, only one study was included (19), with an OR of 0.10 (95% CI: 0.01–0.77, P = 0.03). The direction of effect favored the control group, suggesting that patients in the control group performed significantly better than those in the TD group in this study. As this result is based on a single study, its robustness is limited, and it should be interpreted with caution. The IC group included 2 studies, with an OR of 1.42 (95% CI: 0.48–4.19, P = 0.52), failing to demonstrate a significant advantage of IC over the control group. There was moderate heterogeneity among the studies in this group (I² = 58%, P = 0.12); however, due to the small number of included studies, the reliability of the heterogeneity estimate is limited. RT included 3 studies, with an OR of 1.08 (95% CI: 0.17–6.92, P = 0.93), showing no significant efficacy. Furthermore, there was extremely high heterogeneity among the studies (I² = 93%, P < 0.00001), with marked differences in the direction and magnitude of effects across studies. The overall meta-analysis showed that the pooled OR for all interventions was 0.92 (95% CI: 0.30–2.81, P = 0.89), which was not statistically significant, and overall heterogeneity was high (I² = 87%, P < 0.00001). Subgroup analysis results indicated that differences in efficacy among the three subgroups were not statistically significant (P = 0.08, I² = 61.2%), suggesting that, overall, there were no significant differences in the relative efficacy of the various interventions in this analysis (**Figure 5**).

**Figure 6:**
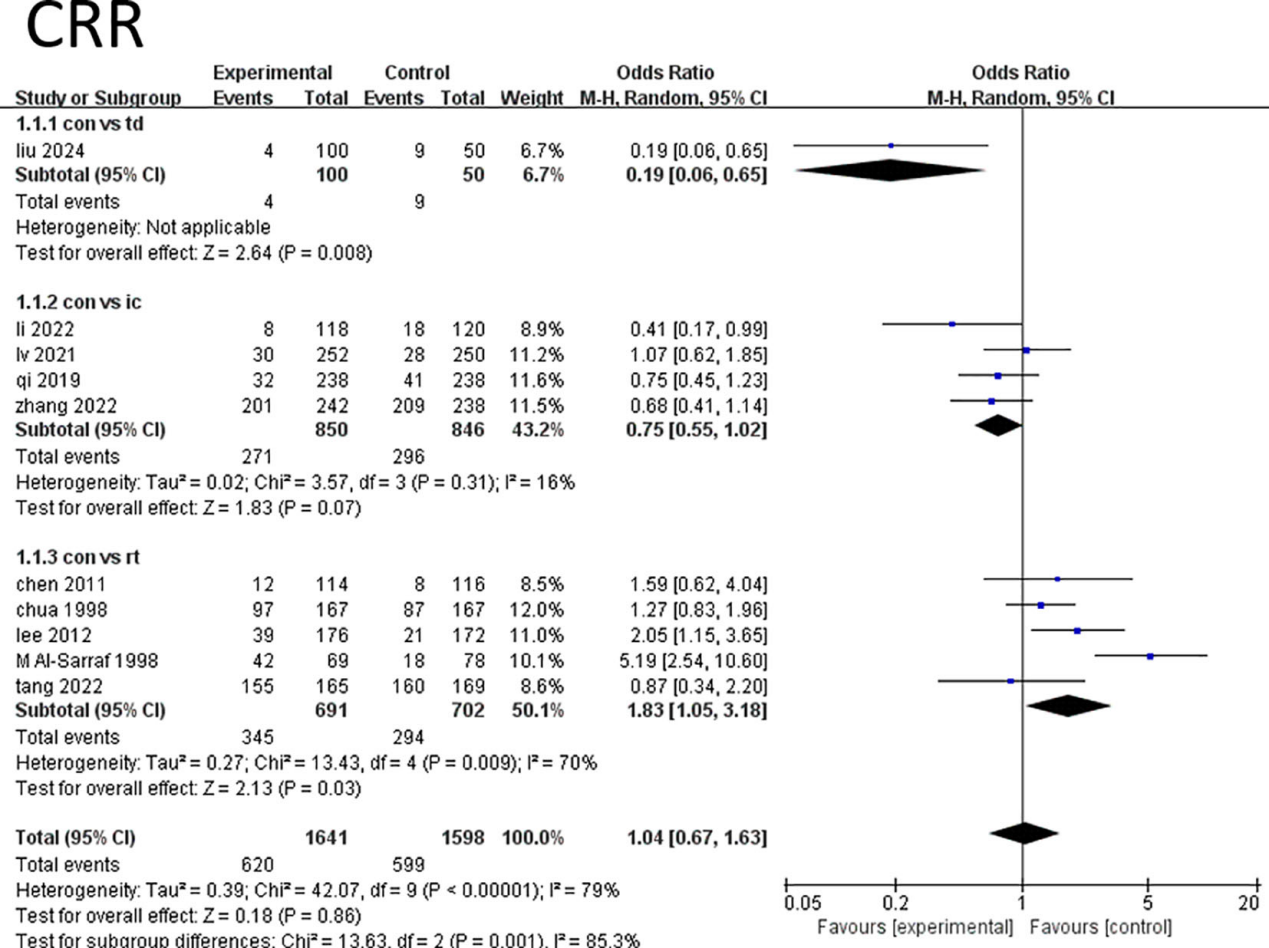
Forest of cumulative recurrence rate.

Meta-analysis results for CRR: A meta-analysis was conducted involving 3,239 participants to systematically evaluate the efficacy of different interventions. **Figure 6** presents the forest plot analysis results for cumulative recurrence rates. This study compared the effects of various interventions versus the control group on cumulative recurrence rates. Results indicate that across three subgroups, targeted drugs (TD) showed a significant trend toward reducing recurrence risk, with a pooled odds ratio of 0.19 (95% CI [0.06, 0.65]), reaching statistical significance (P = 0.008); induction chemotherapy (IC) had a pooled odds ratio of 0.75 (95% CI [0.55, 1.02], P = 0.07), which was not statistically significant but suggested a potentially beneficial direction; while radiotherapy (RT) showed a pooled OR of 1.83 (95% CI [1.05, 3.18], P = 0.03), statistically significant but with an effect direction suggesting this intervention may increase recurrence risk. The overall analysis showed that the combined effect of all interventions was not statistically significant, with a pooled OR of 1.04 (95% CI [0.67, 1.63], P = 0.86). Additionally, substantial heterogeneity was present (I²=79%, P<0.00001), indicating significant variation among studies. Subgroup analysis revealed significant intergroup heterogeneity (I² = 85.3%, P = 0.001), suggesting that differences in intervention types constitute a major source of heterogeneity. In summary, based on current evidence, TD demonstrates the most favorable effect in reducing cumulative recurrence rates, while RT may increase recurrence risk. Future studies should further explore sources of high heterogeneity and clarify the true effects of various interventions.

### 3.5 Network meta-analysis

#### 3.5.1 Network diagram of included studies

**Figure 7:**
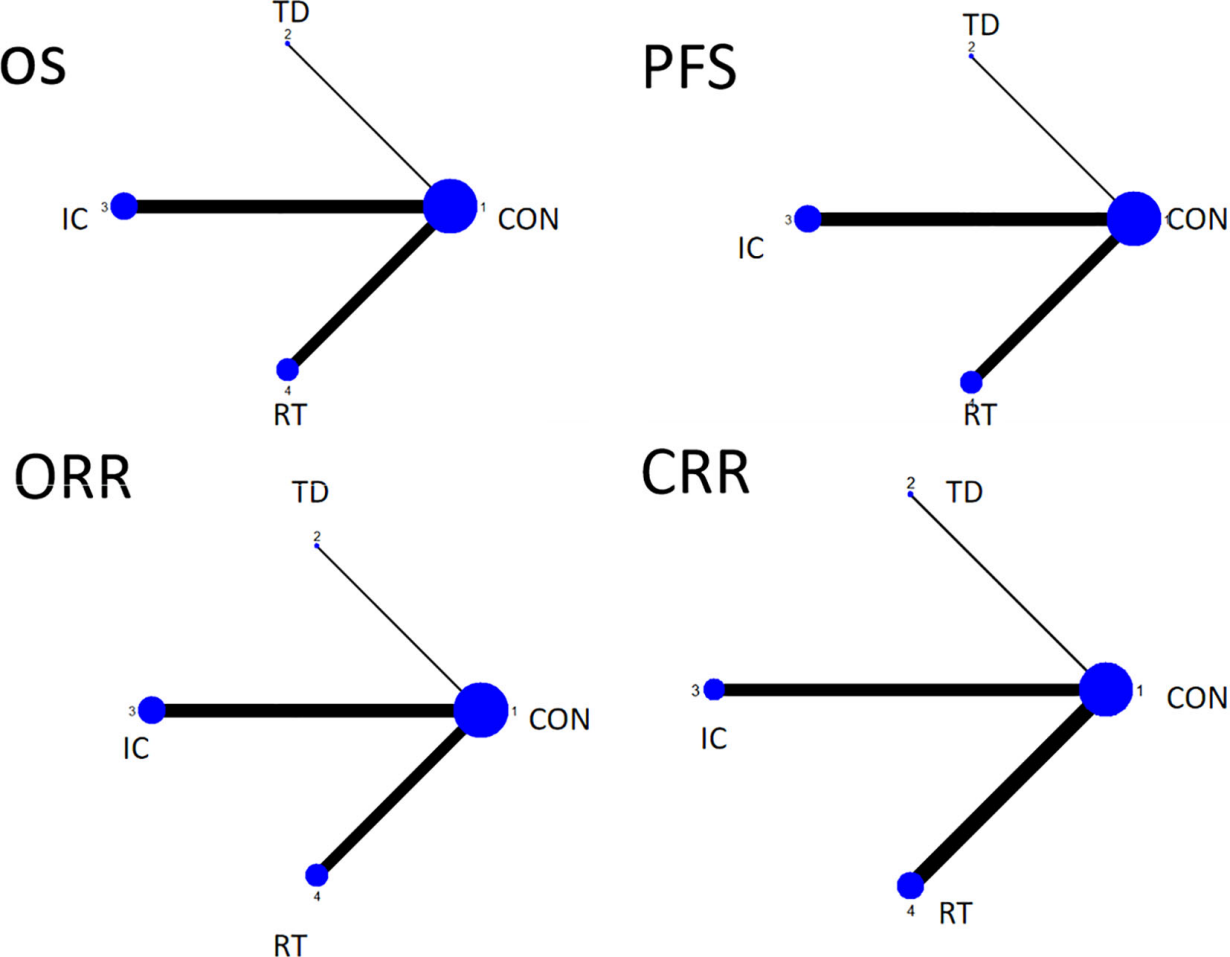
Network plot of outcome indicators.

**Figure 7** presents the NMA network diagram for three drug therapies, evaluating their efficacy. Node size reflects the sample size for each drug therapy, while line thickness indicates the number of studies comparing these interventions. Targeted therapy (TD) and induction chemotherapy (IC) are commonly used interventions, whereas radiotherapy (RT) has fewer studies. **Figure 7** also details the network diagram for outcome measures.

#### 3.5.2 Intervention effects of three treatment approaches

In terms of OS, as shown in **Tables 4** and **5**, the efficacy of the three treatment regimens was ranked as follows: TD (SUCRA = 98.1%), IC (SUCRA = 67.2%), and RT (SUCRA = 0.4%); the SUCRA value for the control group (CON) was 34.3%. The network meta-analysis matrix shows that, compared with CON, both IC (HR = 0.37, 95% CI: 0.02–0.71) and RT (HR = 0.93, 95% CI: 0.37–1.48) demonstrated a trend toward or a significant improvement in OS; the comparison between TD and RT also favored TD (HR = 2.96, 95% CI: 0.67–5.25).

**Table 4:** Ranking the probability of three treatment approaches.

Treatment	Overall survival		Treatment	Progression-free survival		Treatment	Objective response rate		Treatment	Cumulative recurrence rate	
	SUCRA (%)	Rank		SUCRA (%)	Rank		SUCRA (%)	Rank		SUCRA (%)	Rank
CON	34.3	3	CON	33.6	3	CON	56.5	3	CON	36.9	3
TD	98.1	1	TD	82.2	1	TD	8.5	4	TD	98.0	1
IC	67.2	2	IC	59.3	2	IC	71.6	1	IC	64.7	2
RT	0.4	4	RT	24.9	4	RT	63.5	2	RT	0.4	4

**Table 5:** Network meta-analysis matrix of outcome.

Overall survival HR			
TD	-2.03 (-4.31,0.25)	-2.40 (-4.65,-0.15)	-2.96 (-5.25,-0.67)
2.03 (-0.25,4.31)	IC	-0.37 (-0.71,-0.02)	-0.93 (-1.48,-0.37)
2.40 (0.15,4.65)	0.37 (0.02,0.71)	CON	-0.56 (-0.99,-0.13)
2.96 (0.67,5.25)	0.93 (0.37,1.48)	0.56 (0.13,0.99)	RT
Progression-free survival HR			
TD	0.68 (-1.23,2.59)	0.95 (-0.84,2.73)	1.09 (-0.89,3.06)
-0.68 (-2.59,1.23)	IC	0.27 (-0.41,0.94)	0.41 (-0.67,1.49)
-0.95 (-2.73,0.84)	-0.27 (-0.94,0.41)	CON	0.14 (-0.70,0.99)
-1.09 (-3.06,0.89)	-0.41 (-1.49,0.67)	-0.14 (-0.99,0.70)	RT
Objective response rate OR			
IC	-0.24 (-2.66,2.18)	-0.37 (-2.21,1.46)	-2.68 (-6.34,0.98)
0.24 (-2.18,2.66)	RT	-0.14 (-1.71,1.44)	-2.44 (-5.97,1.09)
0.37 (-1.46,2.21)	0.14 (-1.44,1.71)	CON	-2.31 (-5.47,0.85)
2.68 (-0.98,6.34)	2.44 (-1.09,5.97)	2.31 (-0.85,5.47)	TD
Cumulative recurrence rate OR			
TD	3.82 (1.82, 8.02)	5.26 (1.23, 22.44)	10.07 (2.10, 48.30)
0.26 (0.12, 0.58)	IC	1.38 (0.85, 2.23)	2.54 (1.49, 4.33)
0.19 (0.05, 0.77)	0.72 (0.45, 1.16)	CON	1.82 (1.15, 2.88)
0.10 (0.02, 0.48)	0.39 (0.23, 0.67)	0.55 (0.35, 0.87)	RT

Regarding PFS, the efficacy rankings of the three treatment regimens were: TD (SUCRA = 82.2%), IC (SUCRA = 59.3%), and RT (SUCRA = 24.9%); the SUCRA value for the control group (CON) was 33.6%. In the network meta-analysis matrix, none of the pairwise comparisons between the interventions and the control group (CON) reached statistical significance.

Regarding ORR, the efficacy rankings of the three treatment regimens were: IC (SUCRA = 71.6%), RT (SUCRA = 63.5%), and TD (SUCRA = 8.5%); the SUCRA value for the control group (CON) was 56.5%. In the network meta-analysis matrix, the confidence intervals for all pairwise comparisons of the interventions crossed the null line, indicating no statistically significant differences.

Regarding CRR, the efficacy rankings of the three treatment regimens were: TD (SUCRA = 98.0%), IC (SUCRA = 64.7%), and RT (SUCRA = 0.4%); the SUCRA value for the control group (CON) was 36.9%. The network meta-analysis matrix showed that, compared with CON, both TD (OR = 0.26, 95% CI: 0.12–0.58) and RT (OR = 0.39, 95% CI: 0.23–0.67) significantly reduced the cumulative risk of recurrence. Furthermore, TD did not demonstrate a statistically significant advantage over IC (OR = 1.38, 95% CI: 0.85–2.23), and the difference between IC and RT was also not statistically significant (OR = 0.55, 95% CI: 0.35–0.87).

### 3.6 Ranking of intervention effects for outcome indicators

**Figure 8:**
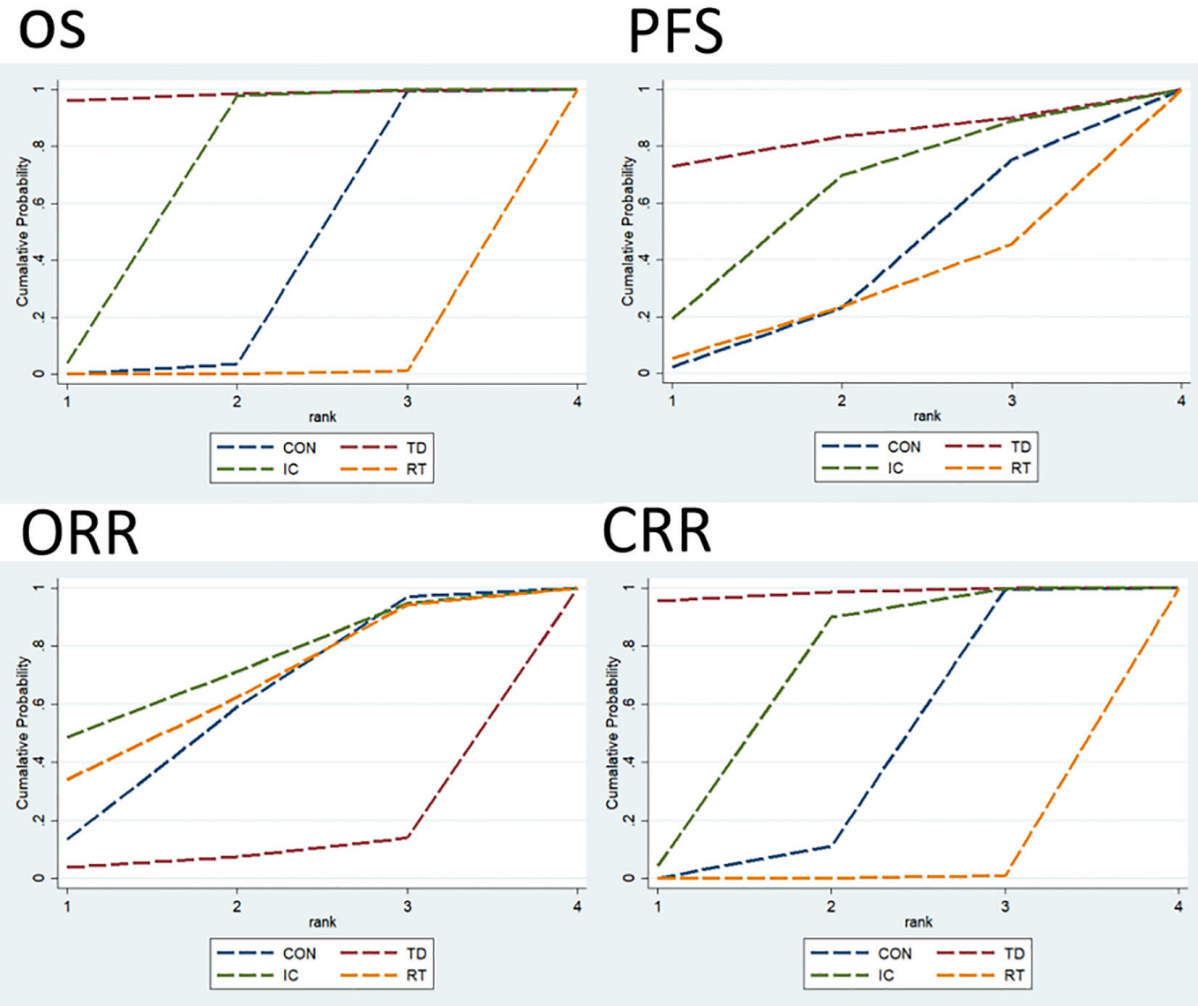
Area under the curve for cumulative ranking probability.

In terms of OS, the SUCRA rankings for the three treatment regimens were as follows: TD (98.1%, ranked first), IC (67.2%, ranked second), CON (34.3%, ranked third), and RT (0.4%, ranked fourth). The results of the network meta-analysis showed that, compared with CON, IC (HR = 0.37, 95% CI: 0.02–0.71) significantly improved OS; whereas the difference between TD and CON was not statistically significant (HR = 2.03, 95% CI: -0.25–4.31). RT showed a trend toward survival benefit compared with CON (HR = 0.93, 95% CI: 0.37–1.48), but its effect size was smaller than that of IC (**Figure 8**).

Regarding PFS, the SUCRA rankings of the three treatment regimens were: TD (82.2%, ranked first), IC (59.3%, ranked second), CON (33.6%, ranked third), and RT (24.9%, ranked fourth). The results of the network meta-analysis showed that none of the pairwise comparisons between the interventions and CON reached statistical significance: TD vs CON (HR = -0.68, 95% CI: -2.59–1.23), IC vs CON (HR = -0.27, 95% CI: -0.94–0.41), and RT vs CON (HR = -0.14, 95% CI: -0.99–0.70). See **Table 4** for details.

Regarding ORR, the SUCRA rankings of the three treatment regimens were: IC (71.6%, ranked first), RT (63.5%, ranked second), CON (56.5%, ranked third), and TD (8.5%, ranked fourth). The results of the network meta-analysis showed that the confidence intervals for all pairwise comparisons of interventions crossed the null line, indicating no statistically significant differences: IC vs CON (OR = 0.24, 95% CI: -2.18–2.66), RT vs CON (OR = 0.14, 95% CI: -1.44–1.71), and TD vs CON (OR = 2.68, 95% CI: -0.98–6.34). See **Table 4** for details.

Regarding CRR, the SUCRA rankings of the three treatment regimens were: TD (98.0%, ranked first), IC (64.7%, ranked second), CON (36.9%, ranked third), and RT (0.4%, ranked fourth). The results of the network meta-analysis showed that, compared with CON, both TD (OR = 0.26, 95% CI: 0.12–0.58) and IC (OR = 0.72, 95% CI: 0.45–1.16) reduced the cumulative risk of recurrence, with the effect of TD being statistically significant. RT also demonstrated a significant reduction in the risk of recurrence compared with CON (OR = 0.39, 95% CI: 0.23–0.67). See **Table 4** for details.

### 3.7 Publication bias and small sample size testing

**Figure 9:**
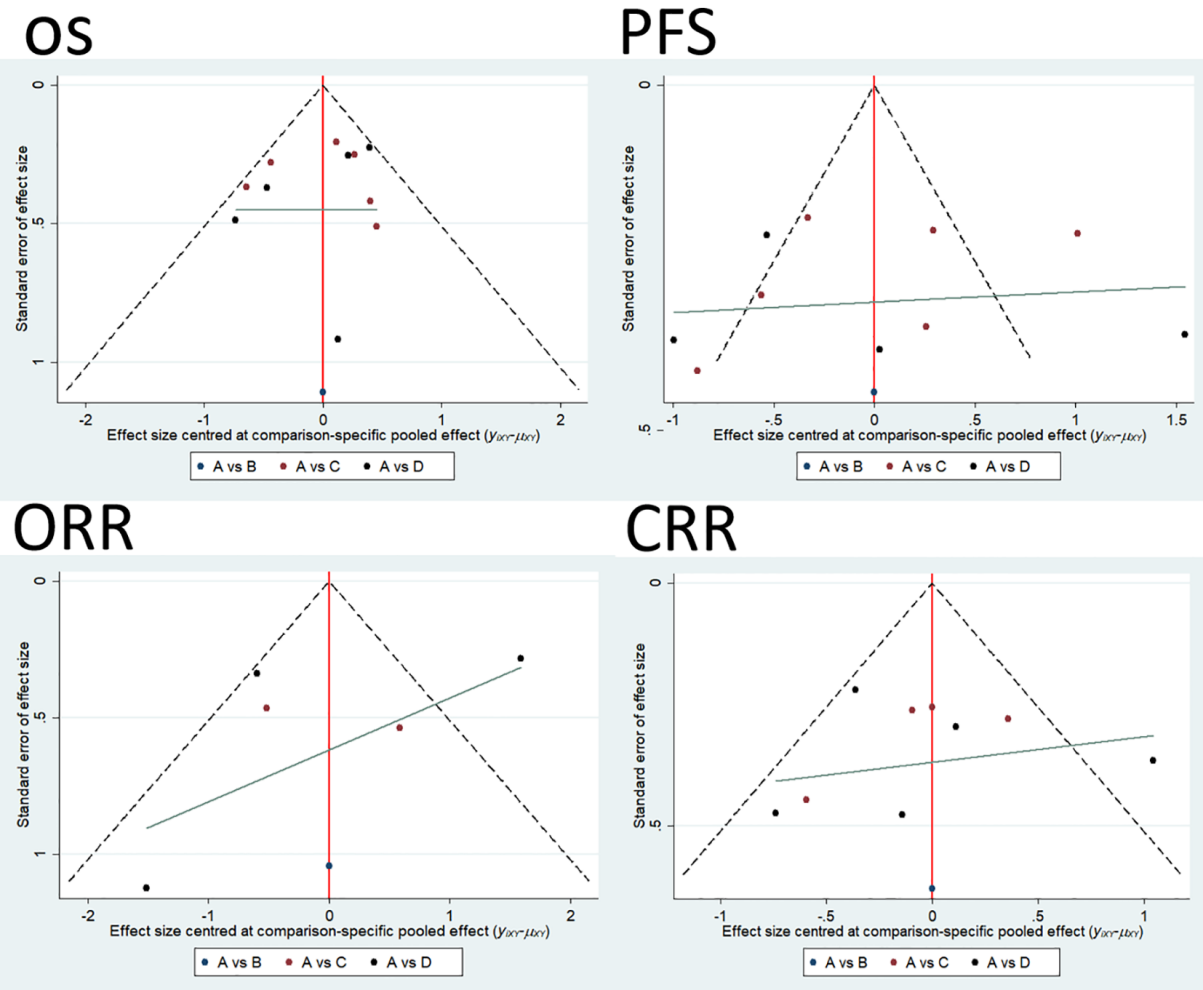
Corrected comparison funnel plot for outcome indicators, A, CON; B, TD; C, IC; D, RT.

For studies included in the network meta-analysis, adjusted comparison funnel plots were used to estimate small-sample effect sizes and assess publication bias. The included studies generally exhibited a symmetrical distribution, with specific details shown in **Figure 9**.

## 4 Discussion

This study systematically compared the effects of IC, TD, and RT on four key outcome measures in patients with nasopharyngeal carcinoma within a unified framework. Regarding OS, TD ranked first in the SUCRA ranking (98.1%), followed by IC (67.2%) (30). However, effect estimates from the network meta-analysis indicated that only IC demonstrated a statistically significant survival benefit compared to CON, whereas TD did not reach statistical significance (31). This finding underscores the importance of interpreting SUCRA rankings in conjunction with effect estimates and their confidence intervals (32). The significant OS benefit of IC is consistent with previous studies supporting the survival benefit of induction chemotherapy, further solidifying its central role in the comprehensive treatment of locally advanced nasopharyngeal carcinoma (33). Although TD ranked first in the SUCRA ranking, its effect estimate lacked statistical significance, indicating that the current evidence is insufficient to confirm a clear survival advantage (1); RT showed no OS benefit and ranked lowest (SUCRA = 0.4%), and in some subgroup analyses, it even showed a trend toward potentially worsened survival outcomes (28). This further supports the current guidelines’ multimodal treatment strategy, which uses concurrent chemoradiotherapy as the cornerstone, combined with IC or TD. The survival benefit of IC is more pronounced in the locally advanced patient population, which is highly consistent with the results of previous studies (34).

In terms of PFS, TD ranked first in the SUCRA ranking (82.2%), followed by IC in second place (59.3%) and RT in fourth place (24.9%) (35). However, the effect estimates from the network meta-analysis showed that none of the pairwise comparisons between the three interventions and CON reached statistical significance (22). This finding suggests that although TD ranked first in the probability-weighted ranking, its effect estimate was accompanied by a wide confidence interval, and the current evidence is insufficient to confirm a clear advantage in PFS (36). It is worth noting that in the direct pairwise meta-analysis, TD was also based on a single study, resulting in a weak evidence base, which further limits the robustness of the conclusions (37). IC demonstrated a significant PFS benefit in the direct pairwise meta-analysis; however, moderate heterogeneity was observed between studies (I² = 68%), suggesting some variation in the efficacy of IC across different studies. Its PFS advantage still requires further validation in a more homogeneous population (38). RT did not demonstrate a significant benefit in terms of PFS, and there was extremely high heterogeneity among studies (I² = 91%), indicating highly inconsistent effects of RT on disease progression across different studies. This may be related to factors such as patient staging, radiotherapy techniques, and whether systemic therapy was combined (39). The above results further support the comprehensive treatment strategy recommended by current guidelines, namely combining systemic therapy with radiotherapy to improve disease control, rather than relying on a single treatment modality (40).

Regarding ORR, IC ranked first in the SUCRA ranking (71.6%), followed by RT (63.5%), TD (8.5%), and CON (56.5%) (41). However, effect estimates from the network meta-analysis showed that none of the pairwise comparisons between the three interventions and the control group (CON) reached statistical significance, with all confidence intervals straddling the line of no effect (24). This result indicates that, although IC ranked first in the probability ranking, the current evidence is insufficient to confirm that any single intervention has a clear advantage in improving ORR (42). It is worth noting that TD had the lowest SUCRA ranking and included only a single study in the direct pairwise meta-analysis; moreover, the effect direction was even in favor of CON, suggesting that this result may be influenced by the characteristics of the single study and should be interpreted with extreme caution (43). High heterogeneity was observed among studies within the IC and RT subgroups, indicating significant differences in the direction and magnitude of ORR effects across studies. This may be related to variations in patient baseline characteristics, tumor stage, treatment regimens, and assessment criteria (44). Overall, ORR, as a measure of short-term efficacy, did not show statistically significant differences among interventions in this analysis. This suggests that treatment selection in clinical decision-making should not rely solely on ORR rankings but should instead be based on a comprehensive evaluation that incorporates survival endpoints (45).

Regarding CRR, targeted therapy demonstrated the most significant reduction in recurrence risk, highlighting its important complementary value in long-term disease control (46); induction chemotherapy also showed a trend toward reduced recurrence risk, whereas radiotherapy alone may increase recurrence risk. These results provide new evidence-based support for the development of clinical relapse prevention strategies; in particular, the integrated use of targeted therapy warrants further exploration for patients at high risk of relapse (47).

Overall, the clinical significance of this study is evident at both the short-term and long-term levels. Regarding short-term outcomes, none of the three regimens demonstrated a statistically significant advantage in ORR over CON, with all confidence intervals crossing the null line. This suggests that short-term response alone should not be the sole criterion for regimen selection in clinical practice. Regarding long-term outcomes, IC was the only intervention to demonstrate a statistically significant OS benefit, while TD showed the most pronounced reduction in CRR. This complementary pattern—IC excelling in prolonging survival and TD excelling in reducing recurrence—provides a clear direction for individualized regimen selection based on treatment goals: IC may be prioritized when the primary objective is to extend long-term survival, whereas TD may offer added value in minimizing recurrence risk, particularly in patients at high risk of relapse. A key strength of this study is that it is the first to use a network meta-analysis to simultaneously compare the relative value of IC, TD, and RT across multiple key outcomes within a single analytical framework. Additionally, the inclusion of RCTs from multiple countries and regions enhances the external generalizability of the findings.

### 4.1 Limitations

This study has several limitations. First, the evidence base for the network meta-analysis primarily uses concurrent chemoradiotherapy as the control group, and direct head-to-head comparisons between different interventions are limited; therefore, some efficacy comparisons rely mainly on indirect evidence. Second, the included studies exhibit significant heterogeneity in terms of radiotherapy techniques, chemotherapy regimens, and patient staging, which may affect the reliability of the pooled estimates. Third, given the clinical differences between locally advanced nasopharyngeal carcinoma treated with curative intent and recurrent/metastatic nasopharyngeal carcinoma treated palliatively, we excluded all trials conducted in a palliative setting. Specifically, of the five initially screened TD trials, four included patients with recurrent or metastatic disease and were therefore excluded. Consequently, only one study (19) remained in the TD group. With only one RCT in the TD group across all outcome analyses (OS, PFS, ORR, and CRR), the precision of both the direct pairwise estimates and the network-derived effect estimates for TD is significantly limited. The wide confidence intervals observed for TD across multiple outcome measures (e.g., OS: HR = 2.03, 95% CI: −0.25–4.31; PFS: HR = −0.68, 95% CI: −2.59–1.23) reflect this inherent uncertainty and require cautious interpretation. Fourth, the included studies span different treatment eras, ranging from the two-dimensional radiotherapy (2D-RT) era of 1998 to the intensity-modulated radiotherapy (IMRT) and immunotherapy era of 2021–2024. Advances in radiotherapy technology and the introduction of targeted drugs may have a significant impact on outcomes, particularly given the extremely high heterogeneity observed in the radiotherapy subgroup (I² = 83%–93%). To assess this impact, we conducted a sensitivity analysis using only studies from the IMRT era. Fifth, the inclusion of Phase II and III trials, as well as a broad range of disease stages from Stage II to IVB, may also be a potential source of heterogeneity. Sixth, it should be noted that when direct pairwise comparisons do not reach statistical significance, the interpretation of relative efficacy based on SUCRA probability rankings is subject to considerable uncertainty. SUCRA rankings may be overinterpreted if the corresponding effect estimates and their confidence intervals are not carefully examined. This consideration is particularly important for the TD group, whose SUCRA rankings (98.1% overall survival, 82.2% progression-free survival, and 98.0% complete response rate) are primarily derived from a single study with limited statistical power, and whose corresponding network effect estimates have wide confidence intervals that straddle the zero line. Future research should focus on conducting more high-quality head-to-head randomized controlled trials in the context of curative treatment to further validate the comparative efficacy of these therapeutic strategies and provide more reliable evidence for optimizing individualized treatment for nasopharyngeal carcinoma.

## 5 Conclusion

This study conducted a network meta-analysis to comprehensively compare the differences in efficacy among three treatment regimens—IC, TD, and RT—for nasopharyngeal carcinoma. The results indicate that IC offers a significant advantage in improving overall survival (OS) and is the most effective strategy for prolonging patient survival. TD shows potential in reducing CRR, highlighting its complementary value in disease control. No statistically significant differences were observed among the three interventions in terms of overall response rate (ORR) and progression-free survival (PFS). Notably, RT did not demonstrate significant benefits across any efficacy endpoints. This study provides evidence-based support for individualized treatment decisions in nasopharyngeal carcinoma and recommends that clinicians select treatment strategies based on specific therapeutic goals. However, current evidence regarding the role of TD in curative treatment is limited to a single randomized controlled trial (RCT), and its efficacy requires further validation through additional prospective studies.

## Data Availability

The original contributions presented in the study are included in the article/**Supplementary Material**. Further inquiries can be directed to the corresponding author.
