# Supplementary material for: Comparative study of three treatment approaches on overall survival and treatment response in nasopharyngeal carcinoma patients: network meta-analysis of RCTs (4221 patients)
Source: Front Oncol. 2026 Jul 2;16:1748308. doi: 10.3389/fonc.2026.1748308 (PMC13372627; doi:10.3389/fonc.2026.1748308)
Supplement: Supplementary file 1 [file DataSheet1.docx]

**1.PubMed search strategy**

(("Nasopharyngeal Carcinoma"[Mesh] OR "Nasopharyngeal Neoplasms"[Mesh] OR nasopharyngeal carcinoma*[Title/Abstract] OR nasopharyngeal cancer*[Title/Abstract] OR nasopharyngeal neoplasm*[Title/Abstract] OR NPC[Title/Abstract]) AND ("Induction Chemotherapy"[Mesh] OR "Molecular Targeted Therapy"[Mesh] OR "Radiotherapy"[Mesh] OR induction chemotherapy[Title/Abstract] OR neoadjuvant chemotherapy[Title/Abstract] OR targeted therapy[Title/Abstract] OR targeted drug*[Title/Abstract] OR molecular targeted therapy[Title/Abstract] OR radiotherapy[Title/Abstract] OR radiation therapy[Title/Abstract] OR chemoradiotherapy[Title/Abstract] OR concurrent chemoradiotherapy[Title/Abstract])) AND ("Randomized Controlled Trial" [Publication Type] OR "Randomized Controlled Trials as Topic"[Mesh] OR randomized[Title/Abstract] OR randomised[Title/Abstract] OR RCT[Title/Abstract])

**2.Sensitivity Analysis**


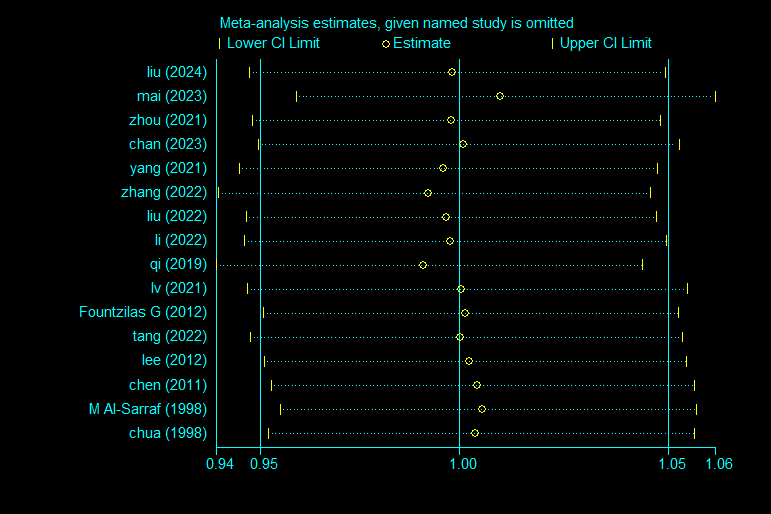


Overall Survival


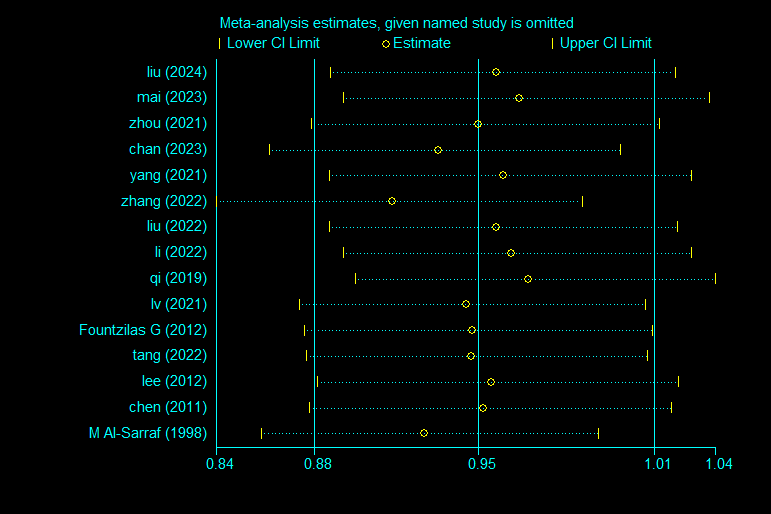


Progression-Free Survival


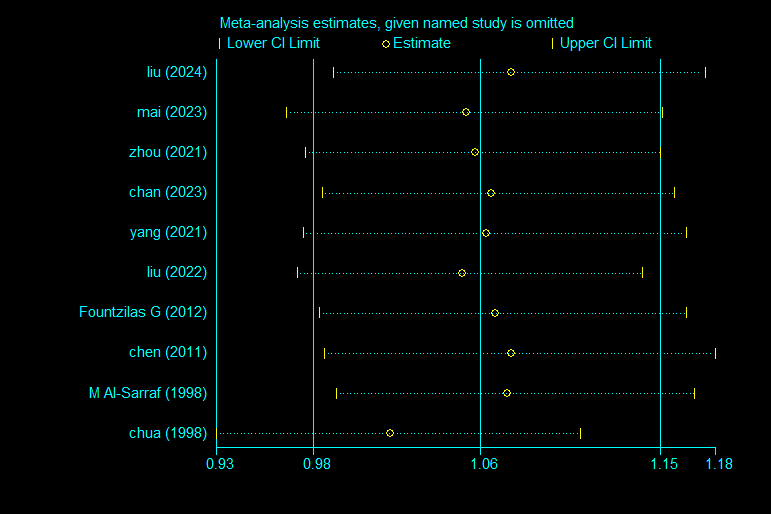


Objective Response Rate


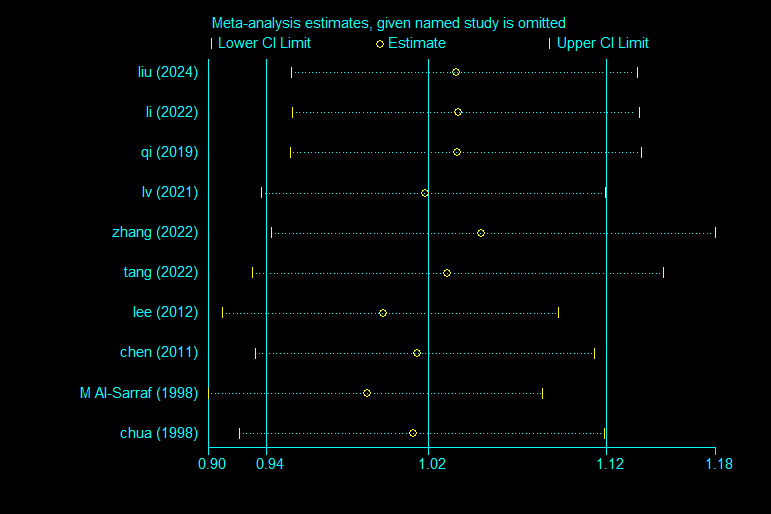


Cumulative Recurrence Rate

局限性方面，部分比较以间接证据为主，直接头对头研究数量有限，且纳入研究在放疗技术、化疗方案及人群分期上存在一定异质性，相关排序需结合具体临床情境谨慎解读。未来研究应聚焦于以生物标志物和分期为导向的优势人群筛选，进一步验证各种治疗策略在精准医学框架下的价值，为优化鼻咽癌个体化治疗提供更高质量的证据支撑。
